# Cardiac Corrected QT Interval Changes Among Patients Treated for COVID-19 Infection During the Early Phase of the Pandemic

**DOI:** 10.1001/jamanetworkopen.2021.6842

**Published:** 2021-04-23

**Authors:** Geoffrey A. Rubin, Amar D. Desai, Zilan Chai, Aijin Wang, Qixuan Chen, Amy S. Wang, Cameron Kemal, Haajra Baksh, Angelo Biviano, Jose M. Dizon, Hirad Yarmohammadi, Frederick Ehlert, Deepak Saluja, David A. Rubin, John P. Morrow, Uma Mahesh R. Avula, Jeremy P. Berman, Alexander Kushnir, Mark P. Abrams, Jessica A. Hennessey, Pierre Elias, Timothy J. Poterucha, Nir Uriel, Christine J. Kubin, Elijah LaSota, Jason Zucker, Magdalena E. Sobieszczyk, Allan Schwartz, Hasan Garan, Marc P. Waase, Elaine Y. Wan

**Affiliations:** 1 Division of Cardiology, Department of Medicine, Vagelos College of Physicians and Surgeons, Columbia University, New York, New York; 2 Department of Biostatistics, Mailman School of Public Health, Columbia University, New York, New York; 3 Vagelos College of Physicians and Surgeons, Columbia University, New York, New York; 4 Department of Medicine, Vagelos College of Physicians and Surgeons, Columbia University, New York, New York; 5 Division of Infectious Disease, Department of Medicine, Vagelos College of Physicians and Surgeons, Columbia University, New York, New York

## Abstract

**Importance:**

Critical illness, a marked inflammatory response, and viruses such as SARS-CoV-2 may prolong corrected QT interval (QTc).

**Objective:**

To evaluate baseline QTc interval on 12-lead electrocardiograms (ECGs) and ensuing changes among patients with and without COVID-19.

**Design, Setting, and Participants:**

This cohort study included 3050 patients aged 18 years and older who underwent SARS-CoV-2 testing and had ECGs at Columbia University Irving Medical Center from March 1 through May 1, 2020. Patients were analyzed by treatment group over 5 days, as follows: hydroxychloroquine with azithromycin, hydroxychloroquine alone, azithromycin alone, and neither hydroxychloroquine nor azithromycin. ECGs were manually analyzed by electrophysiologists masked to COVID-19 status. Multivariable modeling evaluated clinical associations with QTc prolongation from baseline.

**Exposures:**

COVID-19, hydroxychloroquine, azithromycin.

**Main Outcomes and Measures:**

Mean QTc prolongation, percentage of patients with QTc of 500 milliseconds or greater.

**Results:**

A total of 965 patients had more than 2 ECGs and were included in the study, with 561 (58.1%) men, 198 (26.2%) Black patients, and 191 (19.8%) aged 80 years and older. There were 733 patients (76.0%) with COVID-19 and 232 patients (24.0%) without COVID-19. COVID-19 infection was associated with significant mean QTc prolongation from baseline by both 5-day and 2-day multivariable models (5-day, patients with COVID-19: 20.81 [95% CI, 15.29 to 26.33] milliseconds; *P* < .001; patients without COVID-19: −2.01 [95% CI, −17.31 to 21.32] milliseconds; *P* = .93; 2-day, patients with COVID-19: 17.40 [95% CI, 12.65 to 22.16] milliseconds; *P* < .001; patients without COVID-19: 0.11 [95% CI, −12.60 to 12.81] milliseconds; *P* = .99). COVID-19 infection was independently associated with a modeled mean 27.32 (95% CI, 4.63-43.21) millisecond increase in QTc at 5 days compared with COVID-19–negative status (mean QTc, with COVID-19: 450.45 [95% CI, 441.6 to 459.3] milliseconds; without COVID-19: 423.13 [95% CI, 403.25 to 443.01] milliseconds; *P* = .01). More patients with COVID-19 not receiving hydroxychloroquine and azithromycin had QTc of 500 milliseconds or greater compared with patients without COVID-19 (34 of 136 [25.0%] vs 17 of 158 [10.8%], *P* = .002). Multivariable analysis revealed that age 80 years and older compared with those younger than 50 years (mean difference in QTc, 11.91 [SE, 4.69; 95% CI, 2.73 to 21.09]; *P* = .01), severe chronic kidney disease compared with no chronic kidney disease (mean difference in QTc, 12.20 [SE, 5.26; 95% CI, 1.89 to 22.51; *P* = .02]), elevated high-sensitivity troponin levels (mean difference in QTc, 5.05 [SE, 1.19; 95% CI, 2.72 to 7.38]; *P* < .001), and elevated lactate dehydrogenase levels (mean difference in QTc, 5.31 [SE, 2.68; 95% CI, 0.06 to 10.57]; *P* = .04) were associated with QTc prolongation. Torsades de pointes occurred in 1 patient (0.1%) with COVID-19.

**Conclusions and Relevance:**

In this cohort study, COVID-19 infection was independently associated with significant mean QTc prolongation at days 5 and 2 of hospitalization compared with day 0. More patients with COVID-19 had QTc of 500 milliseconds or greater compared with patients without COVID-19.

## Key Points

**Question:**

Is infection with COVID-19 associated with prolonged corrected QT interval (QTc) on electrocardiogram in hospitalized patients?

**Findings:**

In this cohort study of 965 patients with and without COVID-19 infection, multivariable modeling showed that COVID-19 positivity was associated with significant mean QTc prolongation from baseline during a 5-day observation period compared with no significant mean QTc change in patients without COVID-19. A greater proportion of patients with COVID-19 infection had incidence of QTc of 500 milliseconds or greater compared with patients without COVID-19 infection.

**Meaning:**

In this study, COVID-19 infection was associated with significant mean QTc prolongation from baseline, independent of common clinical factors associated with QTc prolongation.

## Teaser

This cohort study evaluates baseline corrected QT interval (QTc) interval on 12-lead electrocardiograms (ECGs) and ensuing changes among patients with and without COVID-19.

## Introduction

COVID-19 has resulted in the death of nearly half a million Americans, with few effective treatment options.^1,2,3^ At the beginning of the pandemic, hydroxychloroquine and azithromycin were studied and frequently prescribed.^4,5^ Because both hydroxychloroquine and azithromycin prolong corrected QT interval (QTc) (via I_Kr_ or *HERG*),^6,7^ QTc interval changes and torsades de pointes (TDP) potential in patients with COVID-19 receiving hydroxychloroquine and/or azithromycin was a focus of multiple single-group studies.^8,9,10,11^

Systemic inflammation prolongs QTc via cytokine-mediated effects on potassium channel expression.^12^ Specific viruses are also associated with QTc prolongation.^13,14,15^ Few analyses have compared QTc intervals between patients with and without COVID-19, an inflammatory viral condition. The present study used multivariable models to investigate which clinical characteristics were associated with prolonged QTc from baseline in patients with COVID-19 and to isolate the independent association of COVID-19 infection with QTc.

## Methods

### Data Collection

Study participants included patients aged 18 years or older who underwent SARS-CoV-2 nasopharyngeal reverse transcriptase–polymerase chain reaction (RT-PCR) testing as well as electrocardiogram (ECG) analysis at Columbia University Irving Medical Center (CUIMC) in New York during inpatient hospitalization from March 1 through May 1, 2020, the first surge of COVID-19. Clinical data, including demographic characteristics, comorbidities, laboratory analyses, ECGs, administrations of concomitant QTc prolonging medications, adverse medication effects, and clinical course after treatment, were obtained from the electronic medical record and deidentified. The study was approved by the CUIMC institutional review board. Informed consent was waived because data were deidentified. This cohort study was reported in adherence to the Strengthening the Reporting of Observational Studies in Epidemiology (STROBE) reporting guideline.

### Patient Selection

The initial baseline cohort included 3050 hospitalized patients tested for COVID-19 at CUIMC between March 1 and May 1, 2020. Patients without ECGs were excluded, and only patients with determinate COVID-19 RT-PCR testing and at least 2 ECGs during a 5-day analysis period were included. The 5-day period included both patients with and without COVID-19 receiving treatment with hydroxychloroquine and/or azithromycin (with proper pharmacy documentation) and patients receiving neither medication. A baseline ECG was defined as the initial ECG within 48 hours of hospital admission. The total number of final participants included in the study was 965 hospitalized patients with and without COVID-19 diagnosed by RT-PCR with a cumulative 2697 ECGs (eFigure 1 in the Supplement). No patients had congenital long QT syndrome.

Patients without COVID-19 received either no treatment or a course of empirical treatment with hydroxychloroquine and/or azithromycin due to delayed diagnostic results of RT-PCR testing during the early pandemic in the context of upper respiratory symptoms initially concerning for COVID-19 infection (eMethods in the Supplement). Some patients without COVID-19 ultimately received an alternative diagnosis, and a number of study participants had repeated negative nasopharyngeal swabs. Patients without COVID-19 were required to never have a single positive RT-PCR test in follow-up. Patients with initial negative swabs who subsequently had a repeated swab that was positive were removed from the study. Thus, patients without COVID-19 included in the study were presumed to have a truly negative RT-PCR test. At CUIMC, the negative predictive value of RT-PCR is 81.3%, which is on par with other centers.^16^ Thus, a rigorous exclusion criterion was implemented in this study to ensure the patients without COVID-19 represented a patient population without infection.

### ECG Analysis

The mean number of ECGs available per patient with and without COVID-19 was similar (eTable 1 in the Supplement). ECGs were manually evaluated by board-certified electrophysiologists and electrophysiology fellows masked to patients’ COVID-19 status. The tracings were evaluated for quality and rejected if there was excessive noise or missing leads impairing interpretation. The QTc intervals were serially evaluated with a standardized protocol by the standard tangent method and excluding U waves, preferably using lead II or V5, and corrected using Bazett and Fridericia formulas.^17,18,19,20^ The QTc is reported using Fridericia in all tables and figures. Further information about ECG analysis appears in the eMethods in the Supplement.

### Statistical Analysis

The distribution of patient baseline characteristics was compared between patients with and without COVID-19 and among patients in the 4 drug treatment groups using χ^2^ tests for categorical variables, analyses of variance for continuous variables, and trend tests for ordinal variables. Biomarker levels were reported as medians and ranges, and log transformations were used in modeling to normalize their distributions when appropriate.

Linear mixed models were used to examine the degree of QTc change during the course of COVID-19 infection (eMethods in the Supplement). Finally, we calculated and compared proportions of patients who had QTc of 500 milliseconds or greater or change in QTc greater than 60 milliseconds at any time during the 5-day treatment period between patients with and without COVID-19 and among patients receiving hydroxychloroquine with azithromycin, hydroxychloroquine only, azithromycin only, and neither drug. Both raw *P* values and adjusted *P* values using Bonferroni corrections are reported. The statistical analyses were conducted in SAS version 9.4 (SAS Institute) and R version 3.6.3 (R Project for Statistical Computing). Statistical significance was set at *P* < .05, and all tests were 2-tailed.

## Results

### Demographic and Clinical Characteristics

Patients with and without COVID-19 were similar in the distribution of age, sex, and race (Table 1). In the cohort of 965 patients, 561 (58.1%) were men, 198 (26.2%) were Black individuals, and 454 (61.9%) were Hispanic/Latino. A total of 191 patients (19.8%) were aged 80 years or older. Overall, 733 patients (76.0%) had COVID-19, and 232 (24.0%) did not. There were significantly more Hispanic/Latino patients with COVID-19 infection than without (380 [67.4%] vs 74 [43.8%]; *P* < .001). Diabetes, hypertension, and moderate or greater kidney dysfunction were common comorbidities in both groups, although hypertension was more prevalent in patients without COVID-19 than those with COVID-19 (129 [55.6%] vs 340 [46.4%]; *P* = .01). Patients with COVID-19 more often had overweight (body mass index [BMI; calculated as weight in kilograms divided by height in meters squared] >25), obesity (BMI >30), or morbid obesity (BMI >35) than those without (530 of 698 [75.9%] vs 130 of 211 [61.6%]; *P* = .007). C-reactive protein (CRP) and ferritin levels were higher in patients with COVID-19 than in those without (CRP: median [range], 18.73 [0.09-50.35] mg/dL vs 9.53 [0.03-30.0] mg/dL; *P* < .001 [to convert to milligrams per liter, multiply by 10]; ferritin: median [range], 1028 [22.6-53 315] ng/mL vs 471.8 [8.8-8863] ng/mL; *P* = .01 [to convert to micrograms per liter, multiply by 1.0]). Patients without COVID-19 were more frequently prescribed concomitant QT prolonging medications than those with COVID-19 (127 [54.7%] vs 266 [36.3%]; *P* < .001). The unadjusted mean (SD) baseline QTc on initial ECG of patients with COVID-19 was significantly lower than that of patients without COVID-19 (424.7 [36.1] milliseconds vs 432.8 [39.0] milliseconds; *P* = .004).

**Table 1.** Baseline Characteristics of Patients With and Without COVID-19

Characteristic	No. (%)			*P* value
	Total (N = 965)	With COVID-19 (n = 733)	Without COVID-19 (n = 232)	
Demographic characteristics				
Age, y				
<50	169 (17.5)	119 (16.2)	50 (21.6)	.33^a^
50-59	157 (16.3)	118 (16.1)	39 (16.8)	
60-69	234 (24.2)	186 (25.4)	48 (20.7)	
70-79	214 (22.2)	163 (22.2)	51 (22)	
≥80	191 (19.8)	147 (20.1)	44 (19)	
Sex				
Women	404 (41.9)	305 (41.6)	99 (42.7)	.78^a^
Men	561 (58.1)	428 (58.4)	133 (57.3)	
Race				
Black	198 (26.2)	148 (26.1)	50 (26.7)	.22^a^
White	261 (34.6)	188 (33.1)	73 (39.0)	
Other^b^	296 (39.2)	232 (40.8)	64 (34.2)	
Not available	210	165	45	NA
Ethnicity				
Hispanic/Latino	454 (61.9)	380 (67.4)	74 (43.8)	<.001^a^
Not Hispanic/Latino	279 (38.1)	184 (32.6)	95 (56.2)	
Not available	232	169	63	NA
GFR stage				
Normal, ≥90 mL/min/1.73 m^2^	207 (21.7)	149 (20.4)	58 (25.8)	.94^c^
Mild, 60-89 mL/min/1.73 m^2^	283 (29.6)	226 (31.0)	57 (25.3)	
Moderate, 30-59 mL/min/1.73 m^2^	265 (27.7)	212 (29.0)	53 (23.6)	
Severe, 15-29 mL/min/1.73 m^2^	99 (10.4)	66 (9.0)	33 (14.7)	
Failure, <15 mL/min/1.73 m^2^	101 (10.6)	77 (10.5)	24 (10.7)	
Not available	10	3	7	NA
BMI				
Underweight, <18.5	28 (3.1)	14 (2.0)	14 (6.6)	.007^c^
Normal, 18.5-24.9	221 (24.3)	154 (22.1)	67 (31.8)	
Overweight, 25.0-29.9	294 (32.3)	241 (34.5)	53 (25.1)	
Obesity, 30.0-35.0	195 (21.5)	158 (22.6)	37 (17.5)	
Morbid obesity, >35.0	171 (18.8)	131 (18.8)	40 (19.0)	
Not available	56	35	21	NA
QT prolonging drugs	393 (40.7)	266 (36.3)	127 (54.7)	<.001^d^
Hypertension	469 (48.6)	340 (46.4)	129 (55.6)	.01^a^
Diabetes	310 (32.1)	233 (31.8)	77 (33.2)	.69^a^
QTc baseline, mean (SD), ms	426.7 (37.0)	424.7 (36.1)	432.8 (39.0)	.004^d^
High-sensitivity troponin, median (range), ng/mL	0.035 (0.006-44.612)	0.032 (0.006-3.563)	0.04 (0.006-44.612)	.003^d^
CRP, median (range), mg/dL	175.6 (0.3-503.5)	187.3 (0.9-503.5)	95.3 (0.3-300)	<.001^d^
Ferritin, median (range), ng/mL	977.8 (8.8-53 315)	1028 (22.6-53 315)	471.8 (8.8-8863)	.02^d^
LDH, median (range), U/L	497 (113-5336)	508 (113-5336)	411 (117-5000)	.32^d^
Lactate, median (range), mg/dL	22.52 (5.41-225.22)	22.52 (5.41-225.22)	25.22 (6.31-198.20)	.007^d^

Abbreviations: BMI, body mass index (calculated as weight in kilograms divided by height in meters squared); CRP, C-reactive protein; GFR, glomerular filtration rate; LDH, lactate dehydrogenase; NA, not applicable; QTc, corrected QT interval.

SI conversions: To convert CRP to milligrams per liter, multiply by 10; ferritin to micrograms per liter, multiply by 1.0; high-sensitivity troponin to micrograms per liter, multiply by 1.0; lactate to millimoles per liter, multiply by 0.111; and LDH to microkatals per liter, multiply by 0.0167.

^a^ Pearson χ^2^ test.

^b^ Patient self-reported as Asian, American Indian, Pacific Islander, or belonging to other racial group.

^c^ Trend test for ordinal variables.

^d^ Linear model analysis of variance.

During hospitalization, 1 patient with COVID-19 infection had TDP. She was in her sixties, was receiving medical therapy for nonischemic cardiomyopathy, and had hypertension, diabetes, and chronic kidney disease. She had QTc of 528 milliseconds in the setting of hypoxemic respiratory failure. After receiving empirical intravenous azithromycin, TDP occurred requiring defibrillation. Potassium was 4.2 mEq/L (to convert to millimoles per liter, multiply by 1.0); magnesium, 1.4 mg/dL (to convert to millimoles per liter, multiply by 0.4114); CRP, 0.002 mg/dL; and high-sensitivity troponin, 42 ng/mL (to convert to micrograms per liter, multiply by 1.0) at admission. After electrolyte repletion and azithromycin withdrawal, TDP resolved.

### Factors Associated With QTc Prolongation at Day 2 and Day 5 of Hospitalization

In Table 2 and the Figure, multivariable analysis performed from day 0 to day 2 of treatment including patients with and without COVID-19 infection showed that patients with severe kidney dysfunction (ie, chronic kidney disease [CKD]) and age 80 years or older had QTc prolongation compared with baseline (severe CKD: difference, 12.20 [SE, 5.26; 95% CI, 1.89 to 22.51] milliseconds; *P* = .02; age ≥80 years: difference, 11.91 [SE, 4.69; 95% CI, 2.73 to 21.09] milliseconds; *P* = .01). Lactate dehydrogenase (LDH) and high-sensitivity troponin level elevation were also significantly associated with QTc prolongation compared with baseline (LDH: difference, 5.31 [SE, 2.68; 95% CI, 0.06-10.57] milliseconds; *P* = .04; high-sensitivity troponin: difference, 5.05 [SE, 1.19; 95% CI, 2.72-7.38] milliseconds; *P* < .001).

**Table 2.** Results of Multivariable Model of QTc From Day 0 Through Day 2

Characteristic (N = 685)^a^	QTc, difference (SE), 95% CI, ms		*P* value^b^
Age, y			
50-59 vs <50	2.97 (4.30) [−5.47 to 11.40]		.49
60-69 vs <50	4.14 (4.02) [−3.74 to 12.01]		.30
70-79 vs <50	4.75 (4.27) [−3.61 to 13.11]		.27
>80 vs <50	11.91 (4.69) [2.73 to 21.09]		.01^c^
Sex			
Male vs female	0.26 (2.65) [−4.93 to 5.44]		.92
Race			
Black vs White	−5.87 (3.74) [13.19 to 1.45]		.12
Other vs White	−2.51 (3.35) [−9.09 to 4.06]		.45
Unknown vs White	−6.43 (3.84) [13.96 to 1.10]		.09
Ethnicity			
Hispanic/Latino vs not specified	−0.63 (3.35) [−7.21 to 5.94]		.85
Not Hispanic/Latino vs not specified	4.49 (4.00) [−3.37 to 12.34]		.26
BMI			
Underweight vs normal	−6.79 (8.49) [−23.43 to 9.85]		.42
Overweight vs normal	0.38 (3.41) [−6.30 to 7.07]		.91
Obesity vs normal	−4.05 (3.71) [−11.32 to 3.22]		.28
Morbid obesity vs normal	−5.74 (4.02) [−13.61 to 2.13]		.15
GFR stage			
Mild CKD vs normal	2.85 (3.58) [−4.17 to 9.87]		.43
Moderate CKD vs normal	2.42 (3.95) [−5.32 to 10.17]		.54
Severe CKD vs normal	12.20 (5.26) [1.89 to 22.51]		.02^d^
Failure CKD vs normal	5.01 (5.48) [−5.73 to 15.75]		.36
QT prolonging medication vs none	−2.49 (2.57) [−7.53 to 2.55]		.33
Hypertension	2.34 (2.83) [−3.20 to 7.87]		.41
Diabetes	2.22 (3.00) [−3.66 to 8.10]		.46
High-sensitivity troponin^e^	5.05 (1.19) [2.72 to 7.38]		<.001
CRP^e^	0.88 (1.39) [−1.85 to 3.61]		.53
Ferritin^e^	−2.85 (1.40) [−5.61 to −0.09]		.04
LDH^e^	5.31 (2.68) [0.06 to 10.57]		.04

Abbreviations: BMI, body mass index; CKD, chronic kidney disease; CRP, C-reactive protein; GFR, glomerular filtration rate; LDH, lactate dehydrogenase.

^a^ A total of 280 patients who did not have consecutive electrocardiograms within 12 hours were excluded.

^b^ The adjusted *P* values using Bonferroni corrections for multiple comparisons within each categorical variable. The multivariable model included COVID-19 status and adjusted for other significant clinical covariates that are associated with QTc, including treatment with hydroxychloroquine and/or azithromycin and other drugs. The results for COVID-19 status appear in the Figure, and the results for treatment are in eFigure 2 in the Supplement.

^c^ Adjusted for multiple comparisons, *P* < .05.

^d^ Adjusted for multiple comparisons, *P* < .10.

^e^ The variable was log transformed while fitting the model.

**Figure.**
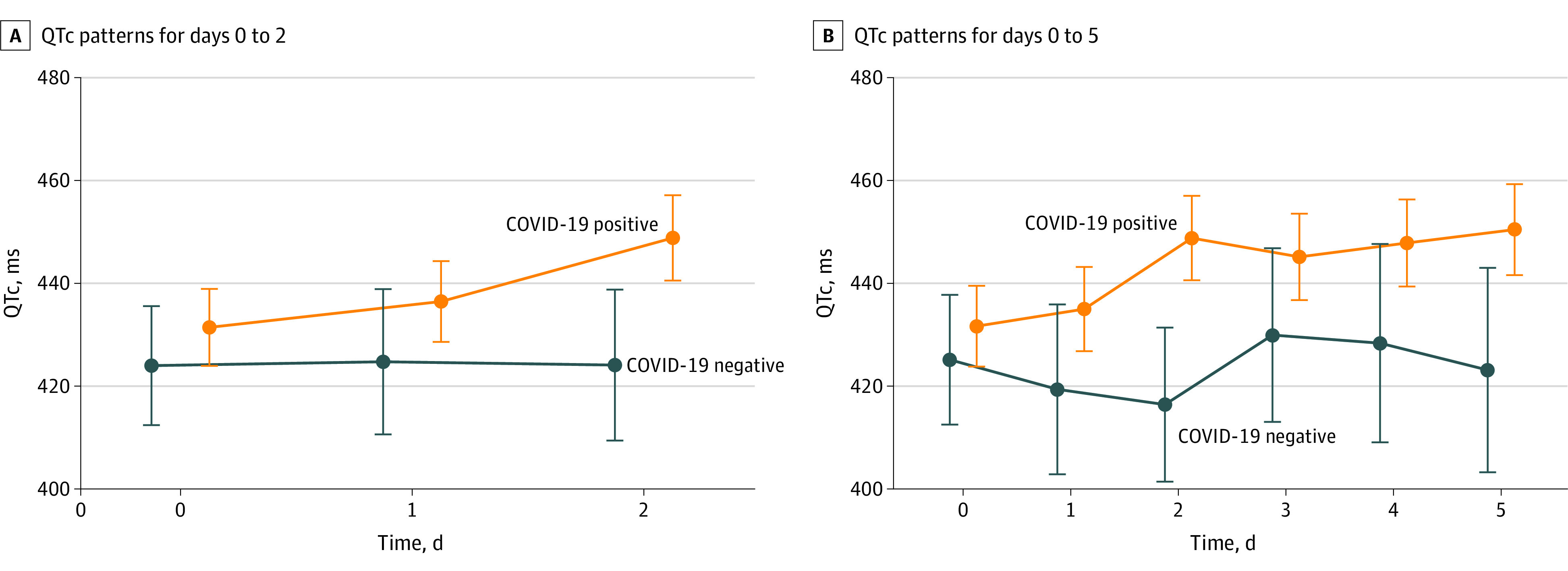
Linear Modeled Corrected QT Interval (QTc) Patterns Between Patients With and Without COVID-19

We compared QTc among patients with and without COVID-19 over 5 days using multivariable analysis. Patients with increased high-sensitivity troponin and kidney failure were more likely to have QTc prolongation from baseline (high-sensitivity troponin: difference, 4.3 [SE, 1.12; 95% CI, 2.13-6.53] milliseconds; *P* < .001; kidney failure: difference, 10.55 [SE, 5.15; 95% CI, 0.46-20.64] milliseconds; *P* = .04). (Table 3). Concomitant use of additional QTc prolonging medications did not have a significant independent association with QTc.

**Table 3.** Results of Multivariable Model of QTc From Day 0 Through Day 5, Excluding Patients Who Received Azithromycin Only

Characteristic	QTc, difference (SE) [95% CI], ms	*P* value^a^
Age, y		
50-59 vs <50	1.48 (4.07) [−6.50 to 9.47]	.72
60-69 vs <50	2.23 (3.79) [−5.19 to 9.65]	.56
70-79 vs <50	1.83 (4.06) [−6.12 to 9.79]	.65
≥80 vs <50	8.26 (4.48) [−0.53 to 17.04]	.07
Sex		
Male vs female	0.47 (2.48) [−4.38 to 5.33]	.85
Race		
Black vs White	−5.69 (3.54) [−12.64 to 1.25]	.11
Other vs White	0.40 (3.15) [−5.77 to 6.57]	.90
Unknown vs White	−2.06 (3.61) [−9.12 to 5.00]	.57
Ethnicity		
Hispanic/Latino vs not specified	−0.12 (3.14) [−6.26 to 6.03]	.97
Not Hispanic/Latino vs not specified	5.12 (3.76) [−2.24 to 12.48]	.17
BMI		
Underweight vs normal	−4.30 (8.17) [−20.31 to 11.70]	.60
Overweight vs normal	1.05 (3.20) [−5.23 to 7.32]	.74
Obesity vs normal	−3.59 (3.48) [−10.40 to 3.23]	.30
Morbid obesity vs normal	−7.83 (3.77) [−15.21 to −0.44]	.04
GFR stage		
Mild CKD vs normal	2.81 (3.33) [−3.73 to 9.34]	.40
Moderate CKD vs normal	4.18 (3.72) [−3.11 to 11.47]	.26
Severe CKD vs normal	9.40 (4.98) [−0.37 to 19.16]	.06
Failure CKD vs normal	10.55 (5.15) [0.46 to 20.64]	.04
QT prolonging medication vs none	−3.73 (2.42) [−8.47 to 1.01]	.12
Hypertension	0.36 (2.67) [−4.87 to 5.59]	.89
Diabetes	0.24 (2.77) [−5.20 to 5.69]	.93
High-sensitivity troponin^b^	4.33 (1.12) [2.13 to 6.53]	<.001
CRP^b^	1.93 (1.34) [−0.71 to 4.56]	.15
Ferritin^b^	−2.39 (1.33) [−4.99 to 0.21]	.07
LDH^b^	3.79 (2.54) [−1.20 to 8.77]	.14

Abbreviations: BMI, body mass index; CKD, chronic kidney disease; CRP, C-reactive protein; GFR, glomerular filtration rate; LDH, lactate dehydrogenase; QTc, corrected QT interval.

^a^ The adjusted *P* values using Bonferroni corrections for multiple comparisons within each categorical variable are all greater than .10 and not shown in the table. The multivariable model included COVID-19 status and adjusted for other significant clinical covariates that are associated with QTc, including treatment with hydroxychloroquine and/or azithromycin and other drugs. The results for COVID-19 status are in the Figure and the results for treatment are in eFigure 2 in the Supplement.

^b^ The variable was log transformed while fitting the model.

Using multivariable analysis, we adjusted for demographic and health characteristics in Table 2 and Table 3. By day 2, patients with COVID-19 had significantly greater mean QTc prolongation from baseline than patients without COVID-19 (mean QTc, 448.83 [95% CI, 440.53-457.13] milliseconds vs 424.1 [95% CI, 409.41-437.78] milliseconds; modeled mean difference, 24.73, [95% CI, 10.74-38.73]; *P* < .001) (Table 4). By day 2, patients with COVID-19 infection had statistically significant mean QTc prolongation of 17.40 (95% CI, 12.65 to 22.16) milliseconds from day 0, (*P* < .001), whereas patients without COVID-19 had QTc increase only of 0.11 (95% CI, −12.60 to 12.81) milliseconds (*P* = .99). There was a significant difference in the modeled QTc temporal patterns over day 0 to day 2 between patients with and without COVID-19 infection (Figure, A).

**Table 4.** Multivariable Model Comparing QTc of Patients With and Without COVID-19

COVID-19 status	Day 0, QTc (95% CI), ms	Day 2, QTc (95% CI), ms	Difference in mean QTC		
			Difference (95% CI), ms	*P *value	Adjusted *P *value^a^
Negative	423.99 (412.42 to 435.56)	424.1 (409.41 to 438.78)	0.11 (−12.60 to 12.81)	.99	>.99
Positive	431.43 (423.94 to 438.91)	448.83 (440.53 to 457.13)	17.40 (12.65 to 22.16)	<.001	<.001^a^
QTc difference between COVID-19 groups	7.44 (−2.67 to 17.54)	24.73 (10.74 to 38.73)	NA	NA	NA
*P* value	.15	<.001	NA	NA	NA
Adjusted *P* value^b^	.45	.002	NA	NA	NA
**COVID-19 status**	**Day 0, QTc (95% CI), ms**	**Day 5, QTc (95% CI), ms**	**Difference (95% CI), ms**	***P *value**	**Adjusted *P *value**^**a**^
Negative	425.14 (412.52 to 437.75)	423.13 (403.25 to 443.01)	−2.01 (−17.31 to 21.32)	.93	>.99
Positive	431.66 (423.8 to 439.52)	450.45 (441.6 to 459.3)	20.81 (15.29 to 26.33)	<.001	<.001
QTc difference between COVID-19 groups	6.52 (−1.51 to 13.16)	27.32 (4.63 to 43.21)	NA	NA	NA
*P* value	.09	.02	NA	NA	NA
Adjusted *P* value^b^	.45	.09^b^	NA	NA	NA

Abbreviations: NA, not applicable; QTc, corrected QT interval.

^a^ The adjusted *P* values using Bonferroni corrections for multiple comparisons in groups of patients with and without COVID-19.

^b^ The adjusted *P* values using Bonferroni corrections for multiple comparisons in Day 0 through Day 2. In the 5-day model, patients treated with azithromycin were excluded.

At day 5, there was significant mean QTc increase compared with baseline level in the patients with COVID-19 vs those without COVID-19 infection (450.45 [95% CI, 441.6 to 459.3] milliseconds vs 423.13 [95% CI, 403.25 to 443.01] milliseconds; mean difference, 27.32 [95% CI, 4.63 to 43.21] milliseconds; *P* = .02) (Table 4). By day 5, patients with COVID-19 had a statistically significant mean QTc prolongation of 20.81 (95% CI, 15.29 to 26.33) milliseconds (*P* < .001), whereas patients without COVID-19 had a nonsignificant mean QTc decrease of 2.01 (95% CI, −17.31 to 21.32) milliseconds; *P* = .93). There was no significant difference in the linear modeled QTc patterns from day 0 to day 5 between patients with and without COVID-19 (Figure, B).

### COVID Infection, QTc of 500 Milliseconds or Greater, and Change in QTc of Greater Than 60 Milliseconds

We analyzed patients with and without COVID-19 infection with QTc of 500 milliseconds or greater or QTc increase of greater than 60 milliseconds (eTable 2 in the Supplement) and 34 of 136 patients (25.0%) with COVID-19 who received neither hydroxychloroquine nor azithromycin over 5 days had QTc of 500 milliseconds or greater compared with only 17 of 158 patients (10.8%) without COVID-19 who had not received hydroxychloroquine nor azithromycin (*P* = .002). Finally, 19 of 255 patients (7.5%) with COVID-19 receiving hydroxychloroquine with azithromycin had QTc of at least 500 milliseconds compared with 0 patients without COVID-19, although this difference was not statistically significant (*P* = .49).

### Treatment Status With Hydroxychloroquine and/or Azithromycin

A high percentage of patients with COVID-19 (68 of 136 [50.0%]) who received neither hydroxychloroquine nor azithromycin were already taking concomitant QTc prolonging agents (eTable 3A and eTable 3B in the Supplement). In the 2-day treatment model, all groups receiving hydroxychloroquine with or without azithromycin had significantly increased mean QTc from baseline (hydroxychloroquine with azithromycin: difference, 11 milliseconds; *P* = .01; hydroxychloroquine alone: difference, 9.7 milliseconds; *P* = .04; azithromycin alone: difference, 15.9 milliseconds; *P* = .03) (eFigure 2 and eTable 4A in the Supplement). The 5-day model revealed similar results (eFigure 2 and eTable 4B in the Supplement). The greatest change in QTc during the 5-day treatment course occurred in the patients receiving hydroxychloroquine with azithromycin, regardless of COVID-19 status (eTable 4 in the Supplement).

Increased cytokine levels may contribute to QTc prolongation. We performed a subset analysis of interleukin 6 (IL-6) levels with QTc maximum in patients with COVID-19 (eFigure 3 in the Supplement). The findings suggested that IL-6 was associated with prolonged QTc, but likely additional factors were involved. To investigate whether patients with COVID-19 and prolonged QTc had increased risk of ventricular arrhythmogenesis, we calculated the ratio between the peak to the end of the T wave (T[p-e]) and QT, an electrocardiographic index of arrhythmogenesis, and found that patients with COVID-19 had significantly longer ratios than those without COVID-19 (eFigure 4 in the Supplement).

## Discussion

To our knowledge, this retrospective observational study at a New York City academic medical center during the peak of the COVID-19 pandemic is the largest to date, evaluating serial QTc changes of nearly 1000 hospitalized patients. The distinctive aspect of our analysis was the inclusion of patients who had negative COVID-19 nasopharyngeal viral swabs but continued to receive hydroxychloroquine and/or azithromycin treatment, which permitted independent analysis of the electrocardiographic association of COVID-19 itself. Vigorous exclusion criteria were applied to minimize the risk of including patients with false-negative COVID-19 tests in the group without COVID-19.

The primary findings were as follows. First, COVID-19 infection was independently associated with increased QTc interval from baseline in multivariable analysis after controlling for confounding clinical characteristics that also prolong QTc, such as age, kidney failure, and concomitant drug treatment (difference, 20.81 milliseconds at day 5; 17.40 milliseconds at day 2). Second, among patients with COVID-19 not receiving treatment with hydroxychloroquine and/or azithromycin, one-quarter had a QTc interval of 500 milliseconds or greater compared with one-tenth of patients without COVID-19 not receiving hydroxychloroquine and/or azithromycin. Third, elevated high-sensitivity troponin and LDH levels, severe kidney injury, and the use of hydroxychloroquine with azithromycin or hydroxychloroquine alone were associated with QTc prolongation from baseline, and patients aged 80 years or older (ie, those most likely to have severe COVID-19 infection and high mortality^21^) had the most marked prolongation of QTc interval.

During the early pandemic in New York City in March 2020, clinical suspicion for COVID-19 was so high in patients presenting to the hospital with respiratory symptoms that empirical treatment with the clinically unproven combination of hydroxychloroquine and azithromycin was frequently used while waiting for the RT-PCR test results. This empirical treatment phenomenon ultimately resulted in a number of patients without COVID-19 at CUIMC receiving full courses of hydroxychloroquine and azithromycin. Therefore, our study design allowed us to identify patients without COVID-19 and compare them with patients with COVID-19 receiving similar drug treatment courses.

Thus far, single-group studies have analyzed the electrocardiographic intervals of patients with COVID-19 rather than including patients without COVID-19. We ensured that patients without COVID-19 in our study never received a positive RT-PCR on repeated testing. The negative predictive value of first-day COVID-19 RT-PCR testing using the CUIMC assay was found to be 81.3%.^16^ A review of 1330 patients across 7 studies showed that the false-negative rate of the RT-PCR at day 3 of symptoms was 20%.^22^

After multivariable adjustment for several factors that concomitantly prolong QTc, infection with SARS-CoV-2 alone was associated with a modeled 27.32 millisecond increase in QTc interval at 5 days compared with patients without COVID-19. While the baseline unadjusted QTc value was significantly longer in patients without COVID-19 than in those with COVID-19 (432.8 milliseconds vs 424.7 milliseconds), after adjustment for other clinical covariates, the baseline QTc was similar between groups and then significantly diverged during the 5-day ECG observation period.

In hospitalized patients, QTc prolongation is usually multifactorial. The severity of each factor—the dose of hydroxychloroquine, the stage of kidney failure, COVID-19 infection, comorbid cardiovascular disease—may be associated with the severity of QTc prolongation, an adverse synergy. In this regard, the modeled QTc increase from baseline level associated with COVID-19 infection (difference at day 5, 20.81 milliseconds; difference at day 2, 17.4 milliseconds) compared with no significant change in the modeled QTc of patients without COVID-19 is a clinically important degree of prolongation from a single variable.

Based on our analysis, patients with elevated levels of high-sensitivity troponin, ferritin, creatinine, and LDH were at higher risk of QTc prolongation from baseline. Nevertheless, the biomarker data reveals some paradoxes. Although the unadjusted mean troponin levels were statistically significantly higher in patients without COVID-19, this group had less QTc prolongation. Although LDH levels were independently associated with QTc prolongation from baseline at day 2, there was no significant difference between baseline LDH levels in patients with and without COVID-19. The troponin and LDH level paradoxes may be explained by clinically insignificant, marginal differences between the 2 groups. Additionally, the biomarkers were associated with relatively small degrees of QTc prolongation (ie, ≤5 milliseconds). This suggests that underlying unmeasured factors, such as sickness severity, stress cardiomyopathy, viral myocarditis, and inflammation, additionally contributed to QTc prolongation. Our data suggest that QTc prolongation of as much as 20 milliseconds was associated with the viral infection.

Other viruses have also been associated with QTc prolongation, including HIV,^13^ HCV coinfection,^14^ and West Nile virus.^15^ HIV-associated inflammation, specifically elevated IL-6 level, is further independently associated with QT prolongation^23^ and prolonged repolarization represented as T wave onset-to-peak duration.^24^ Infection with SARS-CoV-2 may result in cardiac enzyme release, which parallels the systemic inflammatory response marked by elevation of ferritin, LDH, CRP, and IL-6.^25^

Elevations in inflammatory cytokines alone, particularly tumor necrosis factor levels, may increase action potential duration, which would plausibly lead to increased QTc interval on ECG.^26^ In a recent investigation of patients with acute infections, QTc interval prolongation and subsequent normalization paralleled CRP level elevation and subsequent reduction with infection resolution. In the same study, *KCNJ2* potassium channel expression was inversely associated with the inflammatory markers CRP and IL-1.^12^

A true cytokine storm occurs in patients with COVID-19.^27^ Within the inflammatory milieu, IL-6 may play a pivotal role in QTc prolongation and arrhythmogenesis, even more than CRP.^12^ IL-6 has been shown to directly block the *HERG*-K+ channel in ventricular myocardial cells,^28^ inhibit cytochrome p450-3A activity,^29^ and centrally hyperactivate the sympathetic nervous system, which might trigger ventricular arrhythmias.^30^ We performed a subset analysis of IL-6 levels with QTc maximum in patients with COVID-19 (eFigure 3 in the Supplement), which suggested that IL-6 was associated with prolonged QTc, but likely additional factors were involved.

Further clinical analyses shed light on the association between inflammation, QTc prolongation, and arrhythmogenesis. In a study of 5700 patients with COVID-19, median CRP levels were elevated at admission, and a relatively high percentage of patients had QTc of 500 milliseconds or greater.^31^ Another study showed all-cause COVID-19 death was associated with prolonged QTc, which was directly associated with elevated immune-inflammatory markers.^32^ Furthermore, there have been reports of arrhythmic storm in patients with COVID-19 in the setting of massive inflammation.^33,34,35^

There was 1 observed and successfully treated TDP event in our analysis. The patient had QTc of 528 milliseconds, COVID-19 infection, and magnesium level of 1.4 mg/dL and received intravenous azithromycin. The incidence of TDP in the setting of COVID-19 infection in recent studies is similarly low and limited to rare events.^8,9,10,11^

Such ventricular arrhythmogenesis has been associated with the T(p-e).^36^ To further confirm the association of QTc prolongation with COVID-19 infection, we performed analysis of ratios of T(p-e) to QT on day 0 with the 12-lead ECGs of 100 randomly selected patients with and without COVID-19 infection (50 [50.0%] with COVID-19), none of whom were receiving hydroxychloroquine or azithromycin. Patients with COVID-19 had significantly longer ratios (eFigure 4 in the Supplement).

Although not the primary focus of our study, the use of any combination of hydroxychloroquine with azithromycin, hydroxychloroquine alone, or azithromycin alone was associated with significant QTc prolongation. Drug-associated QTc prolongation alone is associated with both increased arrhythmic and nonarrhythmic death, so it is important to study these patients, despite low TDP incidence.^37^ It is more intriguing to note that 25% of patients with COVID-19 (34 patients total) receiving neither drug still had a QTc interval of 500 milliseconds or greater. In patients with COVID-19, especially those receiving hydroxychloroquine with azithromycin, simply checking a baseline pretreatment ECG assessment of QTc may not be sufficient. There have been reports of malignant arrhythmias in patients with COVID-19 receiving hydroxychloroquine with azithromycin.^38,39^ Obtaining 12-lead ECGs to follow the QTc during hospitalization for COVID-19 infection, especially in those older than 80 years, with severe chronic kidney disease, and those with elevated troponin levels is prudent practice.

### Limitations

This study has limitations. It was a retrospective single-center analysis of a diverse ethnic population and includes selection bias and unmeasured confounders that affect the QTc interval and defy multivariable analysis. The results might not apply to a predominantly White population or other centers. Electrolyte levels, ejection fraction, and left ventricular wall thickness were not included in our model. There is potential for interobserver ECG interpretation variation. The baseline ECG QTc prolongation does not uniformly reflect the severity of COVID-19 infection and the time 0 of infection is unknown. Because ECGs were performed more selectively during the peak pandemic, selection bias for cardiac involvement in the patients with COVID-19 may have influenced the QTc interval. A limitation of our modeled statistical design involves multiple comparisons; the repeated comparisons of outcomes may result in false-positive findings when *P* values are between .01 and .05.

The patients without COVID-19 were assumed to have accurate negative viral swabs. There is the potential for false-negative test results despite patients undergoing follow-up testing that reconfirmed COVID-19 negativity. Finally, the sample size of patients without COVID-19 receiving hydroxychloroquine and azithromycin or hydroxychloroquine only was small.

## Conclusions

In this large cohort study of hospitalized patients at a quaternary academic medical institution in an epicenter of the COVID-19 pandemic, we found that COVID-19 infection was independently associated with longer modeled QTc intervals from baseline, and patients at higher risk were 80 years or older, had elevated high-sensitivity troponin, or had significant kidney dysfunction. One-quarter of patients with COVID-19 not receiving hydroxychloroquine or azithromycin experienced QTc of 500 milliseconds or greater.

## Notes

Supplement. **eFigure 1.** Flowchart of Patients With and Without COVID-19 Included and Excluded in Analysis **eMethods.** Supplemental Methods **eTable 1.** Mean ECGs per Treatment Group According to COVID-19 Status **eTable 2.** Patients Who Had QTc of 500 Milliseconds or Greater or Change in QTc of Greater Than 60 Milliseconds at Any Time During the 5-Day Treatment Period **eTable 3.** Baseline Characteristics Comparison of Patients With and Without COVID-19, Subdivided Into Treatment Groups **eFigure 2.** Trends in QTc Changes Over Time in Patients With COVID-19 Receiving Hydroxychloroquine, Hydroxychloroquine With Azithromycin, Azithromycin alone, or Neither Treatment **eTable 4.** Modeled QTc by Treatment Group Including Those Treated with Azithromycin **eFigure 3.** Retrospective Analysis of 56 Patients With COVID-19 With Measured IL-6 Levels and Their QTc Maximum Measurements Over At Least 2 Days **eFigure 4.** T(p-e)/QT Measurements From 12-Lead ECGs From day 0 Hospitalization of Patients With and Without COVID-19 Not Treated With Hydroxychloroquine or Azithromycin
