# Supplementary material for: Cardiac Corrected QT Interval Changes Among Patients Treated for COVID-19 Infection During the Early Phase of the Pandemic
Source: JAMA Netw Open. 2021 Apr 23;4(4):e216842. doi: 10.1001/jamanetworkopen.2021.6842 (PMC8065381; doi:10.1001/jamanetworkopen.2021.6842)
Supplement: Supplement. — eFigure 1. Flowchart of Patients With and Without COVID-19 Included and Excluded in Analysis eMethods. Supplemental Methods eTable 1. Mean ECGs per Treatment Group According to COVID-19 Status eTable 2. Patients Who Had QTc of 500 Milliseconds or Greater or Change in QTc of Greater Than 60 Milliseconds at Any Time During the 5-Day Treatment Period eTable 3. Baseline Characteristics Comparison of Patients With and Without COVID-19, Subdivided Into Treatment Groups eFigure 2. Trends in QTc Changes Over Time in Patients With COVID-19 Receiving Hydroxychloroquine, Hydroxychloroquine With Azithromycin, Azithromycin alone, or Neither Treatment eTable 4. Modeled QTc by Treatment Group Including Those Treated with Azithromycin eFigure 3. Retrospective Analysis of 56 Patients With COVID-19 With Measured IL-6 Levels and Their QTc Maximum Measurements Over At Least 2 Days eFigure 4. T(p-e)/QT Measurements From 12-Lead ECGs From day 0 Hospitalization of Patients With and Without COVID-19 Not Treated With Hydroxychloroquine or Azithromycin [file jamanetwopen-e216842-s001.pdf]

Supplemental Online Content

Rubin GA, Desai AD, Chai Z, et al. Cardiac corrected QT interval changes among patients treated for COVID-19 infection during the early phase of the pandemic. *JAMA Netw Open.* 2021;4(4):e216842. doi:10.1001/jamanetworkopen.2021.6842

eFigure 1. Flowchart of Patients With and Without COVID-19 Included and Excluded in Analysis

eMethods. Supplemental Methods

eTable 1. Mean ECGs per Treatment Group According to COVID-19 Status

eTable 2. Patients Who Had QTc of 500 Milliseconds or Greater or Change in QTc of Greater Than 60 Milliseconds at Any Time During the 5-Day Treatment Period

eTable 3. Baseline Characteristics Comparison of Patients With and Without COVID-19, Subdivided Into Treatment Groups

eFigure 2. Trends in QTc Changes Over Time in Patients With COVID-19 Receiving Hydroxychloroquine, Hydroxychloroquine With Azithromycin, Azithromycin alone, or Neither Treatment

eTable 4. Modeled QTc by Treatment Group Including Those Treated with Azithromycin

eFigure 3. Retrospective Analysis of 56 Patients With COVID-19 With Measured IL-6 Levels and Their QTc Maximum Measurements Over At Least 2 Days

eFigure 4. T(p-e)/QT Measurements From 12-Lead ECGs From day 0 Hospitalization of Patients With and Without COVID-19 Not Treated With Hydroxychloroquine or Azithromycin

This supplemental material has been provided by the authors to give readers additional information about their work.

© 2021 Rubin GA et al. *JAMA Network Open.*

eFigure 1. Flowchart of Patients With and Without COVID-19 Included and Excluded in Analysis

Patients were then subdivided into treatment groups according to HCQ+AZ, HCQ, AZ, or no treatment. Patients were excluded from the study if they had <2 ECGs, inadequate ECG quality, or indeterminate COVID-19 testing. AZ = azithromycin; ECG = electrocardiogram; HCQ = hydroxychloroquine.

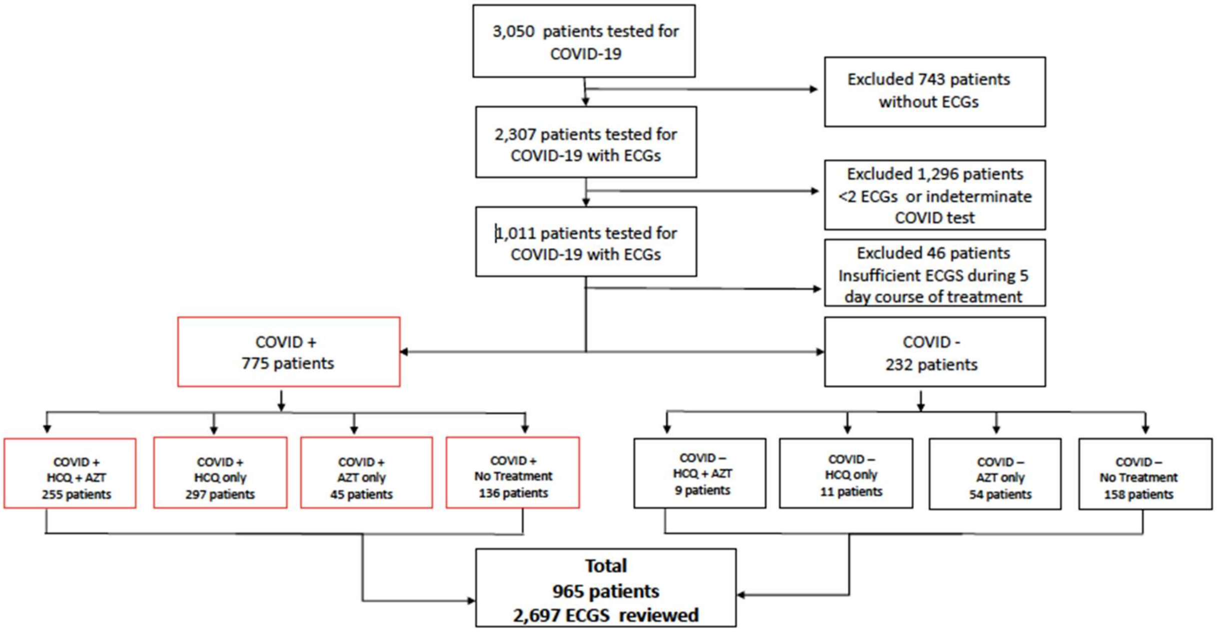

© 2021 Rubin GA et al. *JAMA Network Open.*

eMethods. Supplemental Methods

Patient selection
Although the predominant focus of the analysis was to isolate the COVID-19 effect on QTc prolongation, patients with and without COVID-19 were subcategorized into 4 groups by their treatment status: HCQ+AZ, HCQ alone, AZ alone, or no treatment with HCQ nor AZ (**eFigure 1**). The standard regimen of HCQ was 600mg twice on day 1, then 400mg daily on days 2-5. The standard regimen of AZ was 500mg on day 1, then 250mg daily on days 2-5. By April 3rd, treatment with AZ was no longer recommended at our institution and by April 29th HCQ was removed from our institutional guidelines.

ECG analysis

Markedly prolonged QTc was defined as ≥500ms or an increase of >60ms[21]. When a notched T wave was unable to be distinguished from a superimposed U wave, the QTc measurement was performed on an alternate lead. In the instance of atrial fibrillation, >3 QTc intervals were measured and averaged. In the instance of a paced or wide QRS, 120ms was subtracted from the QRS complex, and that difference was then subtracted from the measured QT interval using the excess correction method[22]. Programs utilized for measurements included MUSE Cardiology Information System (GE Medical Systems, Menomonee Falls, WI) and the Epic Systems (Verona, WI) electronic medical record.

Statistical analysis, multivariable models
The models included the medication course across the different treatment groups, accounting for the correlation of multiple QTc measures at each patient level. Due to the low number of patients receiving AZ after treatment day 3 since empiric AZ treatment fell out of favor in mid-April 2020, two sets of linear mixed models were fit. Multivariable 2-day model analyzed QTc interval changes between COVID+ and COVID- patients from baseline through day 2 of the treatment course, and multivariable 5-day model included analysis across 5 days of treatment but excluded the AZ only group. Both sets of models included patient COVID-19 status, treatments, days, interactions of treatments with days, and interactions of COVID-19 status with days as the key predictors, where the interactions of days with treatments and COVID-19 status evaluated whether the change in the QTc measurement over time varied across the treatment groups or between patients with and without COVID-19, respectively. The models were further adjusted with covariates such as demographics and comorbidity in large multivariable models. The modeled mean QTc levels together with 95% confidence intervals (CIs) were displayed at each day by treatment groups and by COVID-19 status based on the multivariable models, respectively.

© 2021 Rubin GA et al. *JAMA Network Open.*

eTable 1. Mean ECGs per Treatment Group According to COVID-19 Status
Mean number of ECGs for total cohort: 2.79 (95% CI: 2.72-2.87). AZ = azithromycin; CI= confidence interval; ECG = electrocardiogram; HCQ = hydroxychloroquine; n = number of patients.

COVID -	n	Mean (95% CI)
**HCQ + AZ**	9	2.89 (1.91-3.86)
**HCQ**	11	3.00 (2.40-3.60)
**AZ**	54	2.28 (2.13-2.42)
**Neither treatment**	158	2.58 (2.42-2.73)

COVID +	n	Mean (95% CI)
**HCQ + AZ**	255	3.05 (2.90-3.21)
**HCQ**	297	2.96 (2.82-3.09)
**AZ**	45	2.47 (2.14-2.80)
**Neither treatment**	136	2.50 (2.32-2.68)

© 2021 Rubin GA et al. *JAMA Network Open.*

eTable 2. Patients Who Had QTc of 500 Milliseconds or Greater or Change in QTc of Greater Than 60 Milliseconds at Any Time During the 5-Day Treatment Period
AZ = azithromycin; HCQ = hydroxychloroquine; n = number of patients.

Treatment	COVID status	n	QTc 500 ms		>60 ms increase in QTc	
			n (%)	p-value (Adjusted p-value†)	n (%)	p-value (Adjusted p-value†)
**HCQ + AZ**	COVID-	9	0 (0.0%)	1.000 (1.000)	1 (11.1%)	1.000 (1.000)
	COVID+	255	19 (7.5%)		32 (12.5%)	
**HCQ**	COVID-	11	1 (9.1%)	0.490 (1.000)	1 (9.1%)	1.000 (1.000)
	COVID+	297	17 (5.7%)		28 (9.4%)	
**AZ**	COVID-	54	6 (11.1%)	0.109 (0.436)	5 (9.3%)	0.094 (0.282)
	COVID+	45	11 (24.4%)		10 (22.2%)	
**No treatment**	COVID-	158	17 (10.8%)	**0.002** (**0.008**)	10 (6.3%)	0.818 (1.000)
	COVID+	136	34 (25.0%)		10 (7.4%)	

†The adjusted p-values using Bonferroni corrections for multiple comparisons across four treatment groups.

© 2021 Rubin GA et al. *JAMA Network Open.*

eTable 3. Baseline Characteristics Comparison of Patients With and Without COVID-19, Subdivided Into Treatment Groups
Data is presented as # of patients (% of patients). GFR = glomerular filtrate rate, BMI= body mass index, Hs-troponin= high sensitivity troponin, CRP= C-reactive protein, LDH= lactate dehydrogenase

3A. Patients with COVID-19

**Total n=733**	**HCQ + AZ n=255**	**HCQ n=297**	**AZ n=45**	**Neither treatment n=136**	**p-value**
**Age, years**					0.476[1]
<50	46 (18.0%)	42 (14.1%)	11 (24.4%)	20 (14.7%)	
50-59	43 (16.9%)	47 (15.8%)	5 (11.1%)	23 (16.9%)	
60-69	73 (28.6%)	73 (24.6%)	12 (26.7%)	28 (20.6%)	
70-79	52 (20.4%)	71 (23.9%)	9 (20.0%)	31 (22.8%)	
≥80	41 (16.1%)	64 (21.5%)	8 (17.8%)	34 (25.0%)	
**Gender**					0.493[1]
Female	101 (39.6%)	133 (44.8%)	16 (35.6%)	55 (40.4%)	
Male	154 (60.4%)	164 (55.2%)	29 (64.4%)	81 (59.6%)	
**Race**					0.082[1]
Black	59 (30.9%)	48 (20.4%)	7 (20.6%)	34 (31.5%)	
White	53 (27.7%)	84 (35.7%)	15 (44.1%)	36 (33.3%)	
Other	79 (41.4%)	103 (43.8%)	12 (35.3%)	38 (35.2%)	
Not available	64	62	11	28	
**Ethnicity**					**0.044[1]**
Hispanic/Latino	124 (65.6%)	160 (70.8%)	32 (80.0%)	64 (58.7%)	
Not Hispanic/Latino	65 (34.4%)	66 (29.2%)	8 (20.0%)	45 (41.3%)	
Not available	66	71	5	27	
**GFR Stage** (ml/min/1.73m^2)					**<0.001[2]**
Normal (≥90)	48 (18.8%)	74 (24.9%)	10 (22.7%)	17 (12.7%)	
Mild (60-89)	86 (33.7%)	88 (29.6%)	14 (31.8%)	38 (28.4%)	
Moderate (30-59)	74 (29.0%)	85 (28.6%)	14 (31.8%)	39 (29.1%)	
Severe (15-29)	25 (9.8%)	24 (8.1%)	3 (6.8%)	14 (10.4%)	
Failure (<15)	22 (8.6%)	26 (8.8%)	3 (6.8%)	26 (19.4%)	
Not available	0	0	1	2	
**BMI (kg/m^2)**					0.102[2]
Underweight (<18.5)	1 (0.4%)	7 (2.5%)	2 (4.8%)	4 (3.1%)	
Normal Weight (18.5-24.9)	54 (22.0%)	64 (22.6%)	4 (9.5%)	32 (25.2%)	
Overweight (25-29.9)	80 (32.5%)	92 (32.5%)	20 (47.6%)	49 (38.6%)	
Obese (30-35)	62 (25.2%)	61 (21.6%)	8 (19.0%)	27 (21.3%)	
Morbidly obese (>35)	49 (19.9%)	59 (20.8%)	8 (19.0%)	15 (11.8%)	
Not available	9	14	3	9	
**QT prolonging medication**	87 (34.1%)	101 (34.0%)	10 (22.2%)	68 (50.0%)	**0.001[3]**

© 2021 Rubin GA et al. *JAMA Network Open.*

**Hypertension**	106 (41.6%)	145 (48.8%)	22 (48.9%)	67 (49.3%)	0.301[1]
**Diabetes**	82 (32.2%)	98 (33.0%)	10 (22.2%)	43 (31.6%)	0.548[1]
**Hs-Troponin*** median (range)	32.0 (6.0-2784.0)	24.0 (6.0-2107.0)	27.0 (6.0-544.0)	63.0 (6.0-3563.0)	**0.043[3]**
**CRP*** median (range)	219.0 (0.9-457.7)	196.6 (12.3-503.5)	150.6 (3.1-300.0)	135.4 (0.9-300.0)	**<0.001[3]**
**Ferritin*** median (range)	1090.0 (49.1-53315.0)	1082.0 (25.1-49960.0)	820.7(32.1-11555.0)	986.4 (22.6-30518.0)	.152[3]
**LDH*** median (range)	539.5 (113.0-5336.0)	512.5 (176.0-5000.0)	451.0 (157.0-1551.0)	480.5 (154.0-3922.0)	**0.047[3]**

[1]Pearson's Chi-squared test [2]Trend test for ordinal variables [3]Linear Model ANOVA

eTable 3B. Patients without COVID-19.
Data is presented as # of patients (% of patients). GFR = glomerular filtrate rate, BMI= body mass index, Hs-troponin= high sensitivity troponin, CRP= C-reactive protein, LDH= lactate dehydrogenase

**Total n=232**	**HCQ + AZ n=9**	**HCQ n=11**	**AZ n=54**	**Neither treatment n=158**	**p-value**
**Age, years**					0.720[1]
<50	3 (33.3%)	3 (27.3%)	8 (14.8%)	36 (22.8%)	
50-59	2 (22.2%)	2 (18.2%)	9 (16.7%)	26 (16.5%)	
60-69	3 (33.3%)	1 (9.1%)	11 (20.4%)	33 (20.9%)	
70-79	1 (11.1%)	4 (36.4%)	15 (27.8%)	31 (19.6%)	
≥80	0 (0.0%)	1 (9.1%)	11 (20.4%)	32 (20.3%)	
**Gender**					0.528[1]
Female	2 (22.2%)	5 (45.5%)	21 (38.9%)	71 (44.9%)	
Male	7 (77.8%)	6 (54.5%)	33 (61.1%)	87 (55.1%)	
**Race**					0.168[1]
Black	0 (0.0%)	4 (44.4%)	14 (30.4%)	32 (24.8%)	
White	3 (100.0%)	1 (11.1%)	15 (32.6%)	54 (41.9%)	
Other	0 (0.0%)	4 (44.4%)	17 (37.0%)	43 (33.3%)	

© 2021 Rubin GA et al. *JAMA Network Open.*

Not Available	6	2	8	29	
**Ethnicity**					0.252[1]
Hispanic/Latino	4 (80.0%)	3 (33.3%)	20 (50.0%)	47 (40.9%)	
Not Hispanic/Latino	1 (20.0%)	6 (66.7%)	20 (50.0%)	68 (59.1%)	
Not Available	4	2	14	43	
**GFR Stage** (ml/min/1.73m^2)					0.218[2]
Normal (≥90)	2 (22.2%)	3 (27.3%)	12 (22.2%)	41 (27.2%)	
Mild (60-89)	6 (66.7%)	6 (54.5%)	12 (22.2%)	33 (21.9%)	
Moderate (30-59)	1 (11.1%)	0 (0.0%)	13 (24.1%)	39 (25.8%)	
Severe (15-29)	0 (0.0%)	1 (9.1%)	12 (22.2%)	20 (13.2%)	
Failure (<15)	0 (0.0%)	1 (9.1%)	5 (9.3%)	18 (11.9%)	
Not Available	0	0	0	7	
**BMI (kg/m^2)**					0.341[2]
Underweight (<18.5)	0 (0.0%)	1 (9.1%)	6 (12.0%)	7 (4.9%)	
Normal Weight (18.5-24.9)	1 (16.7%)	3 (27.3%)	18 (36.0%)	45 (31.2%)	
Overweight (25-29.9)	2 (33.3%)	3 (27.3%)	9 (18.0%)	39 (27.1%)	
Obese (30-35)	0 (0.0%)	2 (18.2%)	8 (16.0%)	27 (18.8%)	
Morbidly obese (>35)	3 (50.0%)	2 (18.2%)	9 (18.0%)	26 (18.1%)	
Not Available	3	0	4	14	
**QT prolonging medication**	2 (22.2%)	4 (36.4%)	30 (55.6%)	91 (57.6%)	0.118[3]
**Hypertension**	5 (55.6%)	5 (45.5%)	32 (59.3%)	87 (55.1%)	0.857[1]
**Diabetes**	1 (11.1%)	1 (9.1%)	19 (35.2%)	56 (35.4%)	0.150[1]
**Hs-Troponin*** median (range)	12.0 (6.0-16.0)	12.0 (6.0-184.0)	50.0 (6.0-13447.0)	47.0 (6.0-44612.0)	0.930[3]
**CRP*** median (range)	153.1 (47.9-300.0)	205.6 (3.8-293.6)	121.3 (1.4-300.0)	69.0 (0.3-300.0)	0.132[3]
**Ferritin*** median (range)	1332.0 (269.7-2980.0)	429.8 (44.8-4812.0)	266.5 (8.8-4655.0)	494.1 (10.3-8863.0)	0.753[3]
**LDH*** median (range)	458.0 (332.0-834.0)	513.5 (281.0-832.0)	357.0 (125.0-3267.0)	357.0 (117.0-5000.0)	0.957[3]

[1]Pearson's Chi-squared test [2]Trend test for ordinal variables [3]Linear Model ANOVA.

© 2021 Rubin GA et al. *JAMA Network Open.*

eFigure 2. Trends in QTc Changes Over Time in Patients With COVID-19 Receiving Hydroxychloroquine, Hydroxychloroquine With Azithromycin, Azithromycin Alone, or Neither Treatment
Figure 2A shows unadjusted QTc values and Figure 2B shows modeled QTc values. AZ = azithromycin; HCQ = hydroxychloroquine.

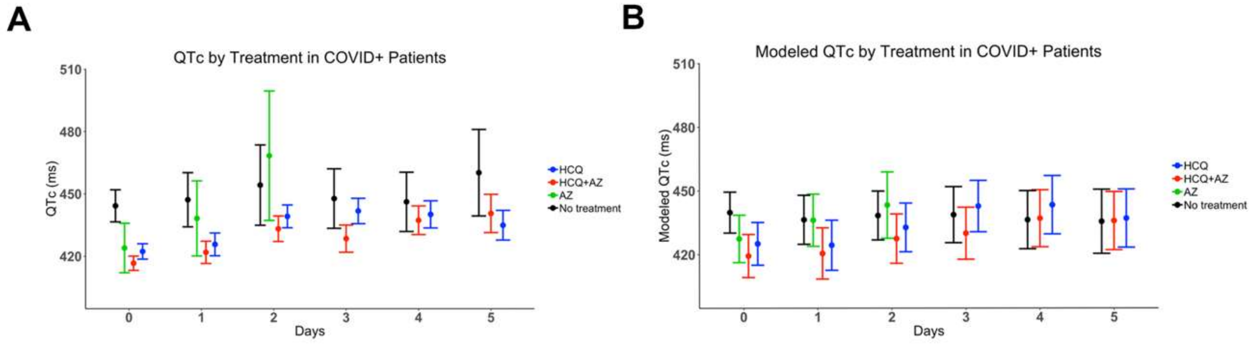

© 2021 Rubin GA et al. *JAMA Network Open.*

eTable 4. **Modeled QTc by Treatment Group Including Those Treated with Azithromycin**

A. Modeled QTc by treatment group from day 0 through day 2 including those treated with AZ. Includes patients with and without COVID-19.

QTc [ms] (95% CI)	Day 0	Day 1	Day 2	p value*
Neither treatment	438.86 (429.54-448.17)	437.69 (426.52-448.87)	438.29 (426.78-449.80)	0.905
HCQ+AZ	419.1 (409.45-428.76)	422.17 (410.79-433.56)	430.14 (418.78-441.50)	0.013
HCQ	425.46 (416.01-434.9)	426.34 (415.42-437.26)	434.12 (423.07-445.17)	0.042
AZ	427.41 (416.25-438.58)	436.19 (423.88-448.51)	443.30 (427.67 – 458.93)	0.025

B. Modeled QTc by treatment group from day 0 through day 5 not including those treated with AZ. Includes patients with and without COVID-19.

QT in ms (95% CI)	Day 0	Day 1	Day 2	Day 3	Day 4	Day 5	p value*
Neither treatment	439.80 (430.16-449.45)	436.40 (424.85-447.95)	438.39 (426.88-449.91)	438.74 (425.54-451.95)	436.35 (422.67-450.04)	435.56 (420.45-450.68)	0.526
HCQ+AZ	419.33 (409.20-429.47)	420.54 (408.48-432.60)	427.50 (415.89-439.11)	430.00 (417.76-442.24)	437.02 (423.59-450.45)	435.90 (422.21-449.59)	0.004
HCQ	425.09 (415.02-435.15)	424.41 (412.62-436.19)	432.77 (421.30-444.23)	442.78 (430.69-454.88)	443.39 (429.65-457.12)	437.06 (423.35-450.77)	0.037

Data presented as average QTc in ms with 95% confidence intervals. *P values represent modeled change in QTc interval from Day 0 to Day 5. AZ = azithromycin; HCQ = hydroxychloroquine.

© 2021 Rubin GA et al. *JAMA Network Open.*

eFigure 3. Retrospective Analysis of 56 Patients With COVID-19 With Measured IL-6 Levels and Their QTc Maximum Measurements Over At Least 2 Days

R^2 is 0.1295

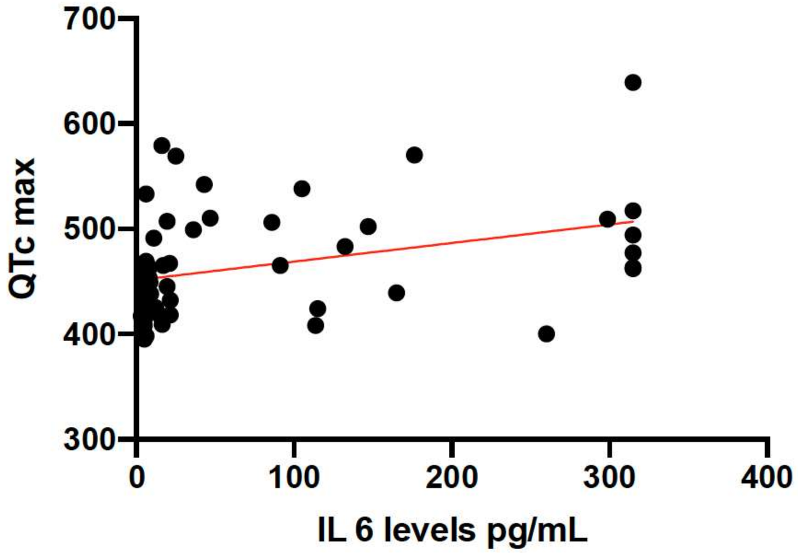

© 2021 Rubin GA et al. *JAMA Network Open.*

eFigure 4. T(p-e)/QT Measurements From 12-Lead ECGs From day 0 Hospitalization of Patients With and Without COVID-19 Not Treated With Hydroxychloroquine or Azithromycin

(avg ± st. dev, 0.26 ± 0.11 vs 0.34 ± 0.14, unpaired t-test p value of 0.0038). We analyzed 50 patients from each group. AZ = azithromycin; HCQ = hydroxychloroquine.

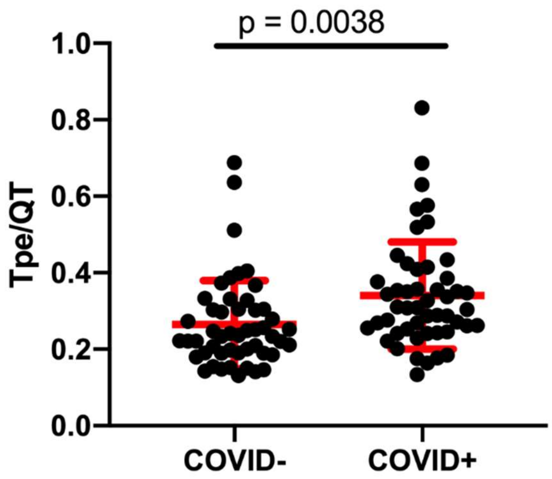

© 2021 Rubin GA et al. *JAMA Network Open.*
